# Empowering Hispanic Multiunit Housing Residents to Advocate for Smokefree Policies: A Randomized Controlled Trial of a Culturally Tailored Fotonovela Intervention

**DOI:** 10.1089/heq.2018.0098

**Published:** 2019-05-02

**Authors:** Jennifer B. Unger, Daniel W. Soto, Angelica Delgado Rendon, Lourdes Baezconde-Garbanati, Tess Boley Cruz

**Affiliations:** Department of Preventive Medicine, University of Southern California, Los Angeles, California.

**Keywords:** Hispanic, multiunit housing, secondhand smoke

## Abstract

**Purpose:** Hispanic residents of multiunit housing (MUH) are disproportionately exposed to secondhand (SHS) and thirdhand tobacco smoke (THS) from neighboring apartment units and common areas. Comprehensive legislation and voluntary policies are needed to protect residents from smoke. We developed a culturally tailored bilingual fotonovela to educate Hispanic residents about SHS and THS and encourage them to talk to their neighbors and landlords about reducing smoke exposure. This article describes a randomized controlled trial of the fotonovela. The objective of the study was to evaluate the effect of the fotonovela on knowledge, attitudes, and behavioral intentions about reducing smoke exposure.

**Methods:** Hispanic MUH residents (*N*=403) completed a survey and were randomly assigned to receive the fotonovela, a text pamphlet, or no materials. They completed a follow-up survey 6 months later.

**Results:** Among the entire sample, there were no significant differences across the three groups in knowledge or attitudes at follow-up. However, when the analyses were restricted to respondents who actually read part or all of the booklets (77% in the fotonovela group and 71% in the text pamphlet group), there were significant differences in two of the six outcome measures; those who read the fotonovela had higher scores on self-efficacy to talk to others about smoke and positive attitudes toward advocacy actions, relative to those who read the text pamphlet.

**Conclusion:** Results indicate that a fotonovela can be an effective tool to empower Hispanic MUH residents to advocate for voluntary smokefree policies, but more efforts are needed to encourage residents to read the materials.

## Introduction

Comprehensive tobacco control efforts in the United States have reduced exposure to tobacco smoke in most public indoor areas, including worksites, restaurants, bars, and hotels.^1^ However, these laws do not include private settings such as the home, which is a primary source of tobacco smoke exposure for children.^2^ Residents of multiunit housing (MUH) are particularly susceptible to involuntary exposure to secondhand (SHS) and thirdhand smoke (THS) from neighboring units or shared outdoor areas.^3,4^ Although most MUH residents prohibit smoking within their own units,^5^ fewer than one-half of MUH residents in the United States live in buildings with smokefree rules, and many report that smokefree rules are unclear or not enforced.^4^ The public health effects of SHS exposure disproportionately affect low-income and racial/ethnic minority populations, who are more likely to live in MUH.^6^ In California, 32% of Hispanics live in MUH, as compared with 22% of non-Hispanic whites.^7^ The smoking prevalence is also higher among Californian adults who live in MUH (18%) than among Californian adults who live in single-family housing (13%).^7^ Thus, Hispanics are more likely to live in MUH, and MUH contains a particularly large concentration of smokers. For MUH residents, living in jurisdictions without smokefree MUH legislation, voluntary policies are another option to protect residents from smoke.

Previous studies^8,9^ have shown that Hispanic MUH residents prefer to live in smokefree environments but are reluctant to ask their neighbors not to smoke or to complain to landlords because of fear of retaliation by smokers, fear of eviction, and cultural values against interfering in other people's business, or challenging elders or authority figures. To address these issues and encourage Hispanic MUH residents to advocate for formal or informal smokefree policies in their buildings, we created a bilingual fotonovela to educate Hispanic apartment residents about SHS and THS. A fotonovela is a small pamphlet similar to a comic book, with photographs instead of illustrations, combined with dialogue bubbles. Fotonovelas typically depict a simple story with a dramatic plot that contains a moral. The goal of the fotonovela was to empower residents to work with their neighbors to find ways to protect residents and their families from smoke. This study is a longitudinal outcome evaluation of the effect of the fotonovela on SHS and THS knowledge and attitudes among Hispanic MUH residents. Previous studies have demonstrated the efficacy of fotonovelas in improving health-related knowledge, attitudes, and behavioral intentions.^10–12^ We evaluated the effectiveness of the fotonovela relative to a text pamphlet about SHS/THS and a nointervention control. We hypothesized that, relative to participants who did not read the fotonovela, participants who read the fotonovela would report (1) higher knowledge about SHS and THS, (2) stronger support for regulations to protect residents from smoke, (3) greater self-efficacy to protect family from smoke and talk to neighbors and landlords about smoke, and (4) more positive attitudes toward advocacy actions.

## Methods

### Development of fotonovela

**FIG. 1.**
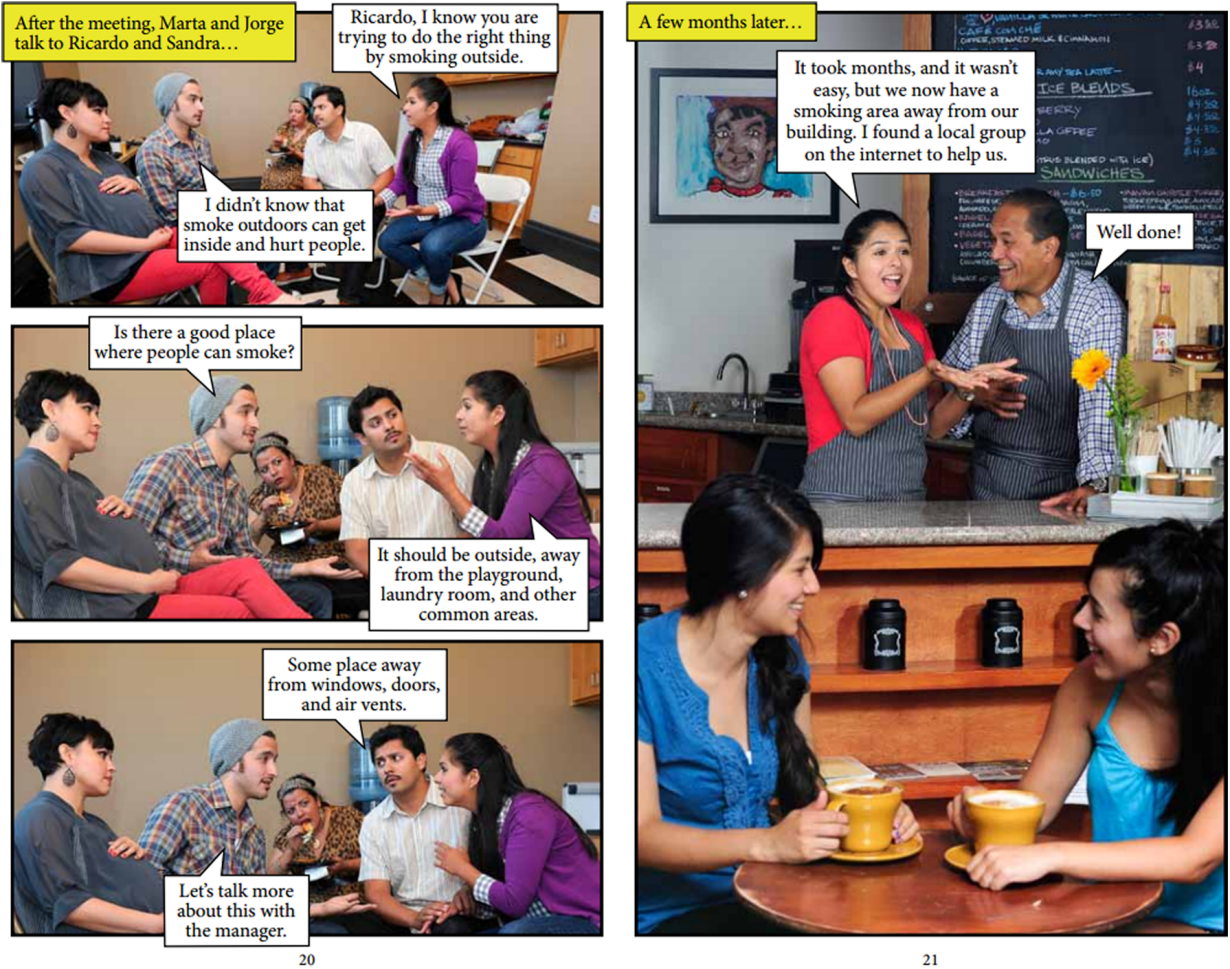
Scenes from the fotonovela. Photo used with permission.

The content of the fotonovela was based on our focus groups of Hispanic MUH residents living in east Los Angeles.^9^ These focus groups revealed that many residents were bothered by SHS/THS but lacked the knowledge, self-efficacy, and empowerment to talk to their neighbors and landlords about the issue. Therefore, we created the fotonovela *Marta on a Mission*, a dramatic story of a Hispanic woman who is bothered by the effects of smoke on her child's asthma and gains the courage to talk to her neighbors. Some of the neighbors initially resist her efforts, but eventually the neighbors all agree that residents have a right to breathe smokefree air and need to protect their children. They decide to designate a smoking area far from the building. The intervention content was informed by social cognitive theory,^13^ which states that improving self-efficacy and modeling the desired behavior should increase the likelihood of the respondent performing the desired behavior. The story was written by a professional scriptwriter at a low literacy reading level, and the photos featured actors in realistic urban Los Angeles locations. The script and layout were pilot tested and modified based on feedback from Hispanic residents. The script was translated and back translated to ensure equivalence between the English and Spanish versions. The fotonovela was printed with the English and Spanish versions back-to-back so readers could choose to read it in their preferred language. Figure 1 shows a page of the fotonovela. A PDF of the fotonovela is available from the authors upon request.

### Sampling and recruitment

Participants were Hispanic adults living in randomly selected MUH buildings in eastern metro Los Angeles. For this study, MUH was defined as a building with at least 10 units, to increase the likelihood that there would be at least one smoker in the building. They were identified using a three-step process: (1) 1250 Census tracts from the 2010 Census were selected that were within a 15-mile radius of east Los Angeles and at least 80% Hispanic; (2) a list was obtained of all apartments within these Census tracts from the Los Angeles County Assessor's Apartment House Listing, and buildings were included if they had at least 10 units, resulting in a sample of 12,344 buildings; and (3) 400 buildings were randomly selected from this list, and 5 units within each building were randomly selected. Only one adult per household and up to five adults per building site could participate. If the selected building was inaccessible (e.g., gated, locked, or no longer being used for MUH), the data collectors approached the next apartment building available to the right, left, or across the street from the original site. Data collectors knocked on the doors of 1272 apartment units. Of those 1272, someone was home and answered the door at 1099 units. Within those 1099 units, 930 had an adult at home who agreed to be screened for eligibility. Residents were eligible to participate if they were Hispanic, ≥18 years of age, and a full-time resident of the unit. Of those who were screened, 279 people did not meet eligibility criteria and 248 declined to participate, resulting in a sample size of 403 adults who provided written informed consent and completed the baseline survey. Recruitment continued until 400 participants had been recruited, based on a power analysis indicating that 400 participants were necessary to detect small to moderate effect.

### Procedure

Respondents completed the pretest survey by responding verbally to questions in their preferred language (English or Spanish). The data collector entered their responses on a tablet computer. After the survey, the computer survey program generated a random number to indicate each respondent's experimental condition: fotonovela, text pamphlet, or control. Respondents randomized to the fotonovela group received a copy of the *Marta on a Mission* fotonovela. Respondents randomized to the text pamphlet group received a bilingual text pamphlet about SHS and THS. Respondents randomized to the control group received no materials. All respondents received a $15 gift card incentive for completing the pretest.

Six months later, participants were recontacted by telephone or in person at their apartments for the follow-up survey; 337 participants completed the follow-up survey (84%). Respondents received a $15 gift card incentive for completing the follow-up survey. All procedures were approved by the University of Southern California Institutional Review Board.

### Measures

The knowledge and attitudes scales were written specifically for this study, because no validated scales about knowledge and attitudes about SHS and THS in MUH exist. These measures were written by a team of experts on tobacco research and health literacy. They were pilot tested and revised for clarity before the baseline survey. Survey development and validation are described elsewhere.^14^

*Knowledge* was measured with 21 factual true/false questions about SHS and THS (e.g., “Thirdhand smoke will go away after the landlord cleans the apartment”). The knowledge score was the number of items that the respondent answered correctly.

The following attitudes were rated on a 4-point scale ranging from 1=strongly disagree to 4=strongly agree. *Favoring rules* was assessed with three questions about whether the respondent favors building-level rules against smoking (e.g., “Would you favor a rule in your building that bans tobacco smoking in all areas, including personal living spaces, such as balconies and patios?”; *α*=0.81). *Self-efficacy to protect family/home from smoke* was assessed with two questions about respondent's confidence in his/her ability to protect his or her home from SHS and THS (e.g., “I feel confident that I can protect my home from secondhand smoke”; *α*=0.70). *Self-efficacy to talk to others about smoke* was assessed with four questions about the respondent's confidence in his/her ability to talk with neighbors and landlords about smoke in the building (e.g., “If my neighbor's smoke bothered me, I feel confident that I could talk to them about it”; *α*=0.73). *Community efficacy* was assessed with three questions about the respondent's confidence that he/she and neighbors could work together to convince the landlord to make smokefree rules and protect the building from SHS and THS (e.g., “I believe that my neighbors and I can protect our building from tobacco smoke”; *α*=0.90). *Advocacy attitudes* were assessed with three questions about whether it is appropriate to confront smokers, organize a meeting, and talk to neighbors about a smoking problem (e.g., “Neighbors should confront smokers directly when secondhand smoke enters their apartments”; *α*=0.75). *Taking action* was assessed with two questions about whether the participant talked to neighbors or the landlord about not smoking in the past 6 months (e.g., “Since the last survey, about 6 months ago, did you talk to your landlord about protecting your apartment from secondhand smoke?”; *α*=0.60).

Variables assessed as potential covariates included age, gender, country of birth (United States vs. other), preferred language, current smoking status, and presence of a smoker in the home.

### Statistical analysis

To assess the equivalence of the three groups at baseline, we used analysis of variance (ANOVA) and chi-square tests to compare the demographic characteristics of the three groups at baseline (age, gender, country of birth, preferred language, current smoking status, and presence of a smoker in the home). After collecting the follow-up data, we compared respondents who were resurveyed successfully with those who were lost to attrition on the same variables. Finally, we conducted ANOVA to compare scores on the outcome variables to compare the three groups at follow-up.

## Results

### Demographic characteristics and smoke exposure at pretest

Pretest surveys were obtained from 403 Hispanic adults who lived in MUH. Most (71%) were female; the mean age was 39.7 years (range=18–93). The majority of participants (69%) were born outside the United States. Slightly over half (59%) completed the survey in Spanish, and 41% completed the survey in English. Although only 22% of the residents lived with a smoker and 97% did not allow smoking inside their units, 80% reported SHS infiltration in their building within the last year.

### Attrition analysis and success of randomization

**FIG. 2.**
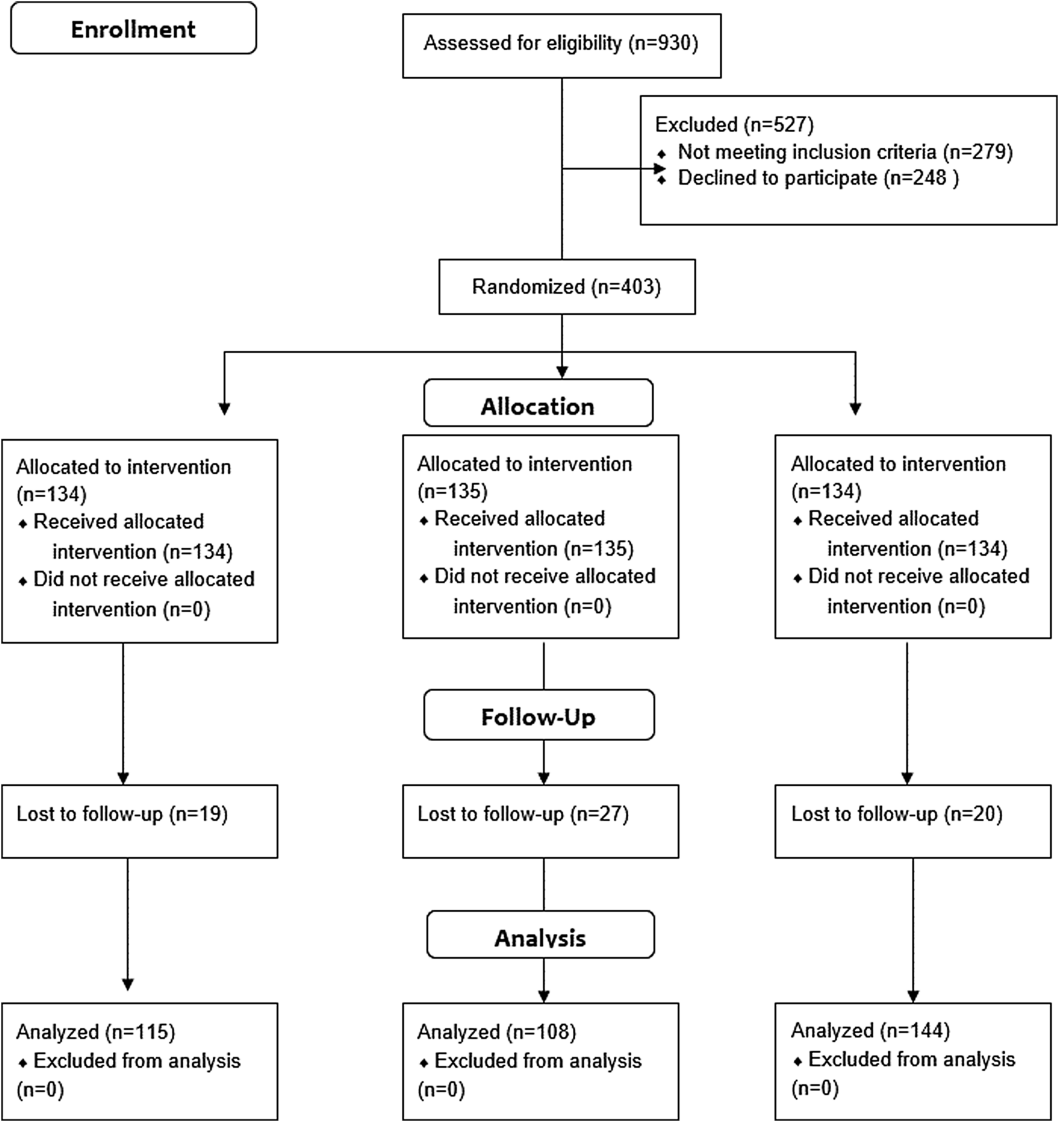
CONSORT diagram.

The CONSORT diagram is shown in Figure 2. We successfully resurveyed 337 participants (84%) at follow-up. Their demographic characteristics and experiences with smoke in MUH are given in Table 1. Compared with those who were followed up successfully, participants who were lost to attrition were significantly younger (mean=35.5 vs. 40.4 years, *p*<0.05), significantly more likely to be male (50% vs. 25%, *p*<0.05), and significantly more likely to have completed the survey in English (53% vs. 38%, *p*<0.05). There was no evidence for differential attrition across treatment conditions.

**Table 1.** Demographic Characteristics of Participants

Variable	Mean or %
Age (years)	40
Female	25%
Born outside the United States	69%
Survey completed in Spanish	62%
Current smoker	9%
Lives with a smoker	19%
At least one child in the home	67%
Allows smoking in individual apartment unit	3%
Someone ever smokes in respondent's unit	9%
Smells smoke in unit at least once per week	25%
Bothered by smell of smoke in home	73%

*N*=337 participants with data at both time points.

At pretest, there were no significant demographic differences across the three randomly assigned groups, indicating that the random assignment was successful. Therefore, subsequent analyses do not control for demographic variables.

### Effectiveness analysis

Among the respondents who had received the fotonovela, 29% read the whole booklet and 48% read part of the booklet. Among the respondents who had received the text pamphlet, 19% read the whole booklet and 52% read part of it (*χ*^2^=3.79, *p*=0.15).

In an analysis of all participants with follow-up data, including those who admitted that they did not read the booklet, there were no significant differences across the three groups (fotonovela, text pamphlet, and control) at follow-up on any of the outcome variables (Table 2). We conducted another comparison of the fotonovela and text pamphlet groups, restricting to participants who read part or all of the booklet. In this analysis, significant differences emerged between the fotonovela group (*N*=86) and the text pamphlet group (*N*=80) on two of the six outcomes, as given in Table 3. Among participants who read part or all of the booklet, those who read the fotonovela showed higher scores on advocacy attitudes and self-efficacy to talk to others about smoke (both *p*<0.05).

**Table 2.** Differences Between the Three Intervention Groups at Follow-Up

Outcome	Fotonovela	Text pamphlet	Control	Significance test
Knowledge	8.31	8.18	8.15	*F*=0.19, ns
Favor rules	1.68	1.52	1.60	*F*=1.10, ns
Self-efficacy to protect	2.10	2.01	2.05	*F*=0.52, ns
Self-efficacy to talk	2.12	1.91	2.00	*F*=2.60, ns
Community efficacy	2.50	2.17	2.31	*F*=1.86, ns
Advocacy	2.06	1.94	2.01	*F*=2.13, ns

**Table 3.** Differences Between the Fotonovela Group and Text Pamphlet Group at Follow-Up, Among Participants Who Read Part or All of the Booklet

Outcome	Fotonovela	Text pamphlet	Significance test
Knowledge	8.35	8.29	*F*=0.03, ns
Favor rules	1.61	1.49	*F*=1.04, ns
Self-efficacy to protect	2.11	2.03	*F*=0.67, ns
Self-efficacy to talk	2.14	1.91	*F*=4.66, *p*<0.05
Community efficacy	2.25	2.15	*F*=1.21, ns
Advocacy	2.04	1.92	*F*=3.76, *p*<0.05

## Discussion

Exposure to SHS and THS in MUH remains a significant public health problem and contributes to health disparities, because minorities and low-income individuals are especially likely to live in MUH. In this study, we created a bilingual culturally targeted fotonovela to raise awareness of SHS and THS and encourage Hispanic residents to talk to their neighbors and landlords about enacting voluntary policies to protect residents from smoke. However, in a randomized controlled trial comparing the fotonovela to a text pamphlet and a control condition, there were no significant differences across the three groups in knowledge, attitudes, or self-efficacy 6 months later.

Part of the overall lack of effect appears to be due to the fact that only a minority of the participants actually read the booklets that they received. Only 29% of the fotonovela group and 18% of the text pamphlet group reported reading the entire booklet. Indeed, when the analyses were restricted to those participants who actually read all or part of the booklet, the fotonovela group showed significantly higher scores on self-efficacy to talk to neighbors and landlords, and positive attitudes toward advocacy actions. However, this decreased our statistical power. This indicates that the fotonovela has the potential to produce positive changes in attitudes and behaviors, but for this to occur, participants need to be motivated to read the fotonovela. The purpose of presenting this information in a fotonovela format was to deliver health education through a dramatic engaging relatable narrative so that participants would be motivated to read it, identify with the characters, and learn effective behaviors through modeling.^15^ However, even if the story is engaging and culturally relevant, a fotonovela alone might not be sufficient to produce attitude and behavior change.

We have conducted several previous studies on the efficacy of health education fotonovelas,^10–12^ and these studies showed significant knowledge and attitude change among participants randomly assigned to read the fotonovela. However, these studies were conducted in the classrooms of community adult schools, and participants were instructed to read the booklet during the class period. Participants are probably more likely to read health education materials when they are instructed to read them in a classroom setting than if they receive the materials from a research staff member at their homes. We did not ask the participants why they did not read the fotonovela or text pamphlet, but it is possible that they were too busy or did not find the material sufficiently relevant or engaging. Future studies should explore ways to make health education materials more engaging, perhaps by turning the narratives into online videos, inviting discussion after they are passed out in person, or creating interactive smartphone apps that guide readers through the novella.

### Limitations

These findings are subject to several limitations. Because there are no validated scales of knowledge and attitudes about SHS and THS in MUH, we developed new measures for this study. These measures demonstrated good internal consistency and reliability, but more reliability and validity testing are warranted. The study did not have sufficient resources to conduct air quality testing in and around the apartments, so we relied on self-reports of SHS and THS exposure. Although we purposely limited the number of residents in each building who could participate to avoid contamination of interventions, it is possible that contamination could have occurred if residents in the same building showed their assigned materials to other residents. Statistical power was limited because many participants did not read the materials.

## Conclusion

The results of this study indicate that a fotonovela can improve knowledge, attitudes, and self-efficacy about avoiding SHS and THS among Hispanic MUH residents. However, the fotonovela can be effective only if people read it. Increased efforts are needed to provide culturally proficient information to Hispanic communities in a format that motivates them to read and use the information. Similar strategies might be effective in other countries, especially among other populations that experience health disparities. Exposure to SHS and THS is a major cause of premature morbidity and mortality worldwide, so effective interventions to help disadvantaged populations avoid smoke exposure could have a significant public health impact.

## Abbreviations Used

ANOVA: analysis of variance
MUH: multiunit housing
SHS: secondhand smoke
THS: thirdhand smoke
